# Germline multigene panel testing of patients with endometrial cancer

**DOI:** 10.3892/ol.2023.13802

**Published:** 2023-04-12

**Authors:** Jan Kral, Sandra Jelinkova, Petra Zemankova, Michal Vocka, Marianna Borecka, Leona Cerna, Marta Cerna, Lukas Dostalek, Petra Duskova, Lenka Foretova, Ondrej Havranek, Klara Horackova, Milena Hovhannisyan, Stepan Chvojka, Marta Kalousova, Marcela Kosarova, Monika Koudova, Vera Krutilkova, Eva Machackova, Petr Nehasil, Jan Novotny, Barbora Otahalova, Alena Puchmajerova, Marketa Safarikova, Jiri Slama, Viktor Stranecky, Ivan Subrt, Spiros Tavandzis, Michal Zikan, Tomas Zima, Jana Soukupova, Petra Kleiblova, Zdenek Kleibl, Marketa Janatova

**Affiliations:** 1 Institute of Medical Biochemistry and Laboratory Diagnostics, First Faculty of Medicine, Charles University and General University Hospital in Prague, Prague 120 00, Czech Republic; 2 Institute of Pathological Physiology, First Faculty of Medicine, Charles University, Prague 120 00, Czech Republic; 3 Department of Oncology, First Faculty of Medicine, Charles University and General University Hospital in Prague, Prague 120 00, Czech Republic; 4 Institute of Biology and Medical Genetics, First Faculty of Medicine, Charles University and General University Hospital in Prague, Prague 120 00, Czech Republic; 5 Center for Medical Genetics and Reproductive Medicine, Gennet, Prague 170 00, Czech Republic; 6 Department of Obstetrics and Gynecology, First Faculty of Medicine, Charles University and General University Hospital in Prague, Prague 120 00, Czech Republic; 7 Laboratory of Molecular Genetics, Hospital Ceske Budejovice, Ceske Budejovice 370 00, Czech Republic; 8 Department of Cancer Epidemiology and Genetics, Masaryk Memorial Cancer Institute, Brno 656 53, Czech Republic; 9 BIOCEV (Biotechnology and Biomedicine Center of The Czech Academy of Sciences and Charles University), First Faculty of Medicine, Charles University, Prague 252 50, Czech Republic; 10 Department of Medical Genetics, Pronatal, Prague 140 00, Czech Republic; 11 Department of Medical Genetics, AGEL Laboratories, AGEL Research and Training Institute, Novy Jicin 741 00, Czech Republic; 12 Department of Paediatrics and Inherited Metabolic Disorders, First Faculty of Medicine, Charles University and General University Hospital in Prague, Prague 120 00, Czech Republic; 13 Department of Biochemistry, Faculty of Science, Charles University, Prague 120 00, Czech Republic; 14 Department of Medical Genetics, Faculty of Medicine in Pilsen, Charles University and University Hospital Pilsen, Pilsen 323 00, Czech Republic; 15 Department of Gynecology and Obstetrics, Bulovka University Hospital and First Faculty of Medicine, Charles University, Prague 180 00, Czech Republic

**Keywords:** uterine malignancies, EC, multigene panel testing, germline mutations

## Abstract

Endometrial cancer (EC) is the most common gynecological malignancy in developed countries. The present study aimed to determine the frequency of germline pathogenic variants (PV) in patients with EC. In this multicenter retrospective cohort study, germline genetic testing (GGT) was performed in 527 patients with EC using a next generation sequencing panel targeting 226 genes, including 5 Lynch syndrome (LS) and 14 hereditary breast and ovarian cancer (HBOC) predisposition genes, and 207 candidate predisposition genes. Gene-level risks were calculated using 1,662 population-matched controls (PMCs). Patients were sub-categorized to fulfill GGT criteria for LS, HBOC, both or none. A total of 60 patients (11.4%) carried PV in LS (5.1%) and HBOC (6.6%) predisposition genes, including two carriers of double PV. PV in LS genes conferred a significantly higher EC risk [odds ratio (OR), 22.4; 95% CI, 7.8-64.3; P=1.8×10^−17^] than the most frequently altered HBOC genes *BRCA1* (OR, 3.9; 95% CI, 1.6-9.5; P=0.001), *BRCA2* (OR, 7.4; 95% CI, 1.9-28.9; P=0.002) and *CHEK2* (OR, 3.2; 95% CI, 1.0-9.9; P=0.04). Furthermore, >6% of patients with EC not fulfilling LS or HBOC GGT indication criteria carried a PV in a clinically relevant gene. Carriers of PV in LS genes had a significantly lower age of EC onset than non-carriers (P=0.01). Another 11.0% of patients carried PV in a candidate gene (the most frequent were *FANCA* and *MUTYH*); however, their individual frequencies did not differ from PMCs (except for aggregated frequency of loss-of-function variants in *POLE*/*POLD1*; OR, 10.44; 95% CI, 1.1-100.5; P=0.012). The present study demonstrated the importance of GGT in patients with EC. The increased risk of EC of PV carriers in HBOC genes suggests that the diagnosis of EC should be included in the HBOC GGT criteria.

## Introduction

Endometrial cancer (EC) is the most common gynecological malignancy in the developed countries (1). Its rate of incidence per 100,000 people in Europe was 32 and in the Czech Republic was 39 in the year 2020 (https://ecis.jrc.ec.europa.eu/). Most EC cases are diagnosed post-menopausally (with a peak incidence between 65–69 years) and in early stages with relatively favorable prognosis (2). EC mortality is approximately four times lower than EC incidence (<20%; www.svod.cz). However, the mortality may vary based on geography and race (3).

Many non-genetic factors modify EC risk. While excess of endogenous estrogens, obesity, insulin resistance, and tamoxifen use increase EC risk, oral contraceptives and sufficient physical activity have protective effects (4).

The risk of EC development is also affected by genetic factors. Germline pathogenic variants (PV) in known EC-predisposition genes are considered the most clinically important [reviewed in (5)]. Germline variants in EC patients were studied by several next generation sequencing (NGS) based studies, dominantly using limited gene panels (21–84 genes) (6–15). These studies reported variable prevalence of germline variants in EC patients ranging from 4.5 to 23%. Majority of hereditary EC cases are associated with Lynch syndrome (LS; also known as hereditary nonpolyposis colorectal cancer), which is caused by germline PV in mismatch repair genes (MMR; *MLH1, MSH2, MSH6, PMS2*, and structural alterations of 3′ end of *EPCAM*) (16). Guidelines for clinical follow-up of carriers of germline PV in LS genes include specific management of increased EC risk. Modest increase of EC risk has been suggested in *BRCA1* and *BRCA2* PV carriers (most notably the serous-like EC subtype), and other hereditary breast and ovarian cancer genes (HBOC; *ATM, BARD1, BRCA1, BRCA2, BRIP1, CDH1, CHEK2, NF1, PALB2, PTEN, RAD51C, RAD51D, STK11, TP53*) (17). Other noteworthy candidate EC-predisposition genes include e.g. *POLD1* and *POLE* (18). Germline missense PV affecting proofreading capabilities of *POLE/POLD1* are associated with increased EC risk as a part of polymerase proofreading-associated polyposis, but the importance of germline *POLD1*/*POLE* truncating variants remains rather elusive (18). Importantly, the genetic basis of most EC cases has not been explained yet as the diagnosis itself is not a criterion for germline genetic testing unless fulfilling LS criteria (5).

We aimed to evaluate germline genetic background of 527 patients with uterine tumors to identify genes associated with EC risk in our population, and to evaluate clinicopathological features in germline PV carriers.

## Materials and methods

### Patients

For this retrospective cohort study, we collected 527 patients with uterine malignancies diagnosed at nine Czech health care centers (General University Hospital in Prague, Masaryk Memorial Cancer Institute, AGEL Laboratories, Gennet, GHC Genetics, University Hospital Pilsen, Pronatal, Palacky University Olomouc) and the Bank of Clinical Samples (First Faculty of Medicine). The full list of all participating institutions is provided in the Table SI. Patients were enrolled between 2011–2021 and were Caucasians of the Czech origin. The clinicopathological characteristics (Table I) revealed that endometrial cancers (EC; 89.7%) were the dominant type of collected uterine malignances, therefore the whole cohort of patients with uterine malignancies will be hereafter referred to as ‘EC patients’. Deficient MMR, microsatellite instability and *MLH1* hypermethylation statuses were not available. We divided patients according to national indication criteria for germline genetic testing of LS and/or HBOC patients:

Breast cancer or ovarian cancer (C50/C56)-national indication criteria for germline genetic testing [HBOC criteria; cancer diagnoses (C##) correspond to the International Classification of Diseases 10; available at https://icd.who.int/browse10/2019/en#/C00-C97]. Personal history: i) patient is diagnosed with C50 <45 years or <50 years, if family history is unknown; ii) patient has bilateral C50 with the age of diagnosis of the first one <50 years and of both <60 years; iii) patient is diagnosed with triple negative C50 ≤60 years; iv) patient is a male diagnosed with C50; v) patient is diagnosed with either C56, C57 or C48; vi) patient has a duplicity od C50 and C25 regardless of age. Family history: i) patient and two relatives are diagnosed with C50; ii) patient and one relative are diagnosed with C50 <50 years or both C50 <60 years (patient included); iii) patient and a direct relative (parent, sibling, child, alternatively mother or father's sister) are diagnosed with either ovarian cancer, fallopian tube or primary peritoneal tumor, triple negative C50/medullar C50, male relative diagnosed with C50, pancreatic cancer, prostate cancer with Gleason score ≥7 or primary metastatic C61.

Colorectal cancer or EC-national indication criteria for germline genetic testing (LS criteria): i) Age of diagnosis <50 years; ii) proven microsatellite instability <60 years; iii) patient has a concurrent diagnosis linked to LS (colorectal cancer, stomach cancer, pancreatic cancer, ovarian cancer, small intestine cancer, ureter cancer, renal pelvis cancer, bile tract cancer, glioblastoma); iv) patient and one first degree relative have diagnoses linked to LS <50 years; v) patient and two second degree relatives have diagnoses linked to LS regardless of the age of diagnosis; and vi) patients with colorectal cancer and more than ten adenomas/polyps.

Of all patients 151/527 (28.7%) met only LS genetic testing criteria, 16/527 (3.0%) met only HBOC criteria, and 82/527 (15.6%) met both these criteria. A total of 278/527 (52.7%) patients would not be indicated for germline genetic testing.

The study was approved by Ethics Committees of participating institutions. Written consent for the research analysis was obtained from all participants. Clinicopathological information was collected during genetic counselling or retrieved from patients' record.

Two sets of population-matched controls (PMC) were used for comparisons with analyzed EC patients. First, used as a reference for genetic variant prioritization, included 777 non-cancer volunteers aged >60 years that were analyzed identically with EC patients as described previously (19). Second group, used in case-control analyses, included 1662 PMC analyzed as described previously (20). Briefly, the unselected controls (1,170 males and 492 females; median age 57 years, range 18–88 years) were unrelated individuals analyzed by whole-exome sequencing by National Center for Medical Genomics (https://ncmg.cz/) for various noncancer conditions.

### Genetic testing using panel NGS

Genomic DNA was isolated from peripheral blood collected at the time of enrollment in each respective center. DNA samples were analyzed by NGS using a custom-designed CZECANCA panel as described previously (21) with minor modifications reflecting recent technological updates. These modifications included a new probe synthesis HyperDesign (Roche) improving target coverage for all 226 genes (the sequence capture panel development is shown in detail on the panel web page: http://www.czecanca.cz/eng/panel.html, and full list of genes targeted in this project is described in Table SII). Further modifications included usage of cheaper and faster enzymatic fragmentation replacing ultrasound DNA fragmentation, preparation of DNA libraries using recently introduced KAPA HyperPlus Library Preparation kit (Roche; according to the manufacturer's instruction) and Illumina NextSeq500 sequencing. Resulting NGS data were processed by an in-house bioinformatics pipeline as we described previously (21). Briefly, SAM files were generated from FASTQ using NovoAlign v2.08.03 (http://www.novocraft.com/products/novoalign/) and transformed into BAM by Picard tools v1.129 (https://broadinstitute.github.io/picard/). The Genome Analysis Toolkit v3.8.1 (https://software.broadinstitute.org/gatk/) (22) was used to prepare variant-call format, annotated by SnpEff v4.3 (http://pcingola.github.io/SnpEff/). Identification of medium size indels was performed by Pindel v0.2.5a7 (http://gmt.genome.wustl.edu/packages/pindel/) and copy number variations (CNV) were detected using CNV kit v0.7.4 (https://pypi.python.org/pypi/CNVkit).

All 226 analyzed genes were divided into 19 known EC-predisposition genes described by NCCN guidelines or reviewed by Spurdle *et al* (5) and 207 other ‘candidate’ genes. Five genes associated with LS (*MLH1, MSH2, MSH6, PMS2, EPCAM*) and 14 genes associated with HBOC (*ATM, BARD1, BRCA1, BRCA2, BRIP1, CDH1, CHEK2, NF1, PALB2, PTEN, RAD51C, RAD51D, STK11, TP53*) were considered as the EC-predisposition genes. The remaining 207 candidate cancer-susceptibility genes included those that have been episodically associated with EC predisposition (incl. *APC, MUTYH, NBN, POLD1, POLE*; Table SII) (5).

### Variant prioritization

Genetic variants found in patients were filtered, excluding variants: i) with low sequencing quality (q<150); ii) with a high minor allele frequency (MAF >0.001) in population databases (gnomAD https://gnomad.broadinstitute.org/, Exome Sequencing Project https://evs.gs.washington.edu/EVS/, 1000 Genomes Project http://www.internationalgenome.org/) (23–25) unless classified as pathogenic/likely pathogenic (P/LP) in the ClinVar database (https://www.ncbi.nlm.nih.gov/clinvar/) (26); iii) present with frequency higher than 0.5% in a group of 777 PMC, except for variants with P/LP ClinVar classification; iv) in untranslated region, intronic outside of consensus splice sites, synonymous and insertion/deletions not resulting in a frameshift unless classified as pathogenic/likely pathogenic (P/LP) in the ClinVar database; v) classified as benign/likely benign in ClinVar with at least two-star rating; vi) low risk variants in *BRCA2* (c.9976A>T; p.Lys3326Ter) and in *CHEK2* (c.470T>C; p.Ile157Thr).

Resulting set of variants was evaluated according to the ACMG (American College of Medical Genetics) recommendations (27). Variants mentioned in ClinVar as a single submitter or with a conflicting interpretation of pathogenicity were categorized as variants of uncertain significance (VUS). Whole gene duplication and truncating variants localized in the last exon were considered VUS, unless they were classified as P/LP in ClinVar. All PV were inspected in Integrative Genomics Viewer or confirmed using Sanger sequencing or multiplex ligation-dependent probe amplification analysis (MRC Holland). Confirmed PV were submitted to ClinVar database.

### Statistical analysis

The frequencies of PV in EC patients were compared with the frequencies of PV in a group of 1662 unselected PMC. Odds ratios (OR) with 95% confidence intervals (CI) were calculated for EC patients carrying found germline PV using 2×2 contingency table. The χ^2^ or Fisher's exact tests were used for the calculation of P-values (considered significant when P<0.05). Differences in age at diagnosis were analyzed by one-way ANOVA followed by Tukey-Kramer's test. Statistical analysis was performed using the R language v4.1.

## Results

### Germline PV in patients with uterine malignances

We performed germline genetic testing in 527 Czech EC patients including 249 individuals fulfilling LS, HBOC, LS + HBOC indication criteria and 278 individuals not fulfilling any criteria for germline genetic testing. Germline PV were significantly more frequent in patients (118/527; 22.4%) than in population-matched controls (290/1662; 17.4%; P=0.011).

### Germline PV in EC-predisposition genes

PV were found in 12 (out of 19 tested) EC-predisposition genes (Table II). Frequency of these variants was more than four-times higher in EC patients (60/527; 11.4%; Table SIII) than in PMC (46/1662; 2.8%; P=9.7×10^−16^). PV in LS genes were found in 27/527 (5.1%) patients (half of them were *MSH6* PV carriers) and in 4/1662 (0.25%) controls and they represented the strongest genetic risk factor for EC development (OR=22.4, P=1.8×10^−17^). Interestingly, no *PMS2* PV were observed among patients. *BRCA1, BRCA2* and *CHEK2* were the most frequently mutated HBOC genes, their PVs conferred significantly increased EC risk for female carriers. However, this risk was lower in comparison to LS genes (ranging from high EC risk in *BRCA2* to moderate EC risk in *BRCA1* and *CHEK2*, respectively). PV in the remaining 11 HBOC genes were not identified or did not differ significantly from PMC (Table II). Two carriers harbored coincidental mutations in *MLH1/BRCA1* and *MSH2*/*ATM*, respectively.

### Indication criteria for identification of PV carriers

**Figure 1.**
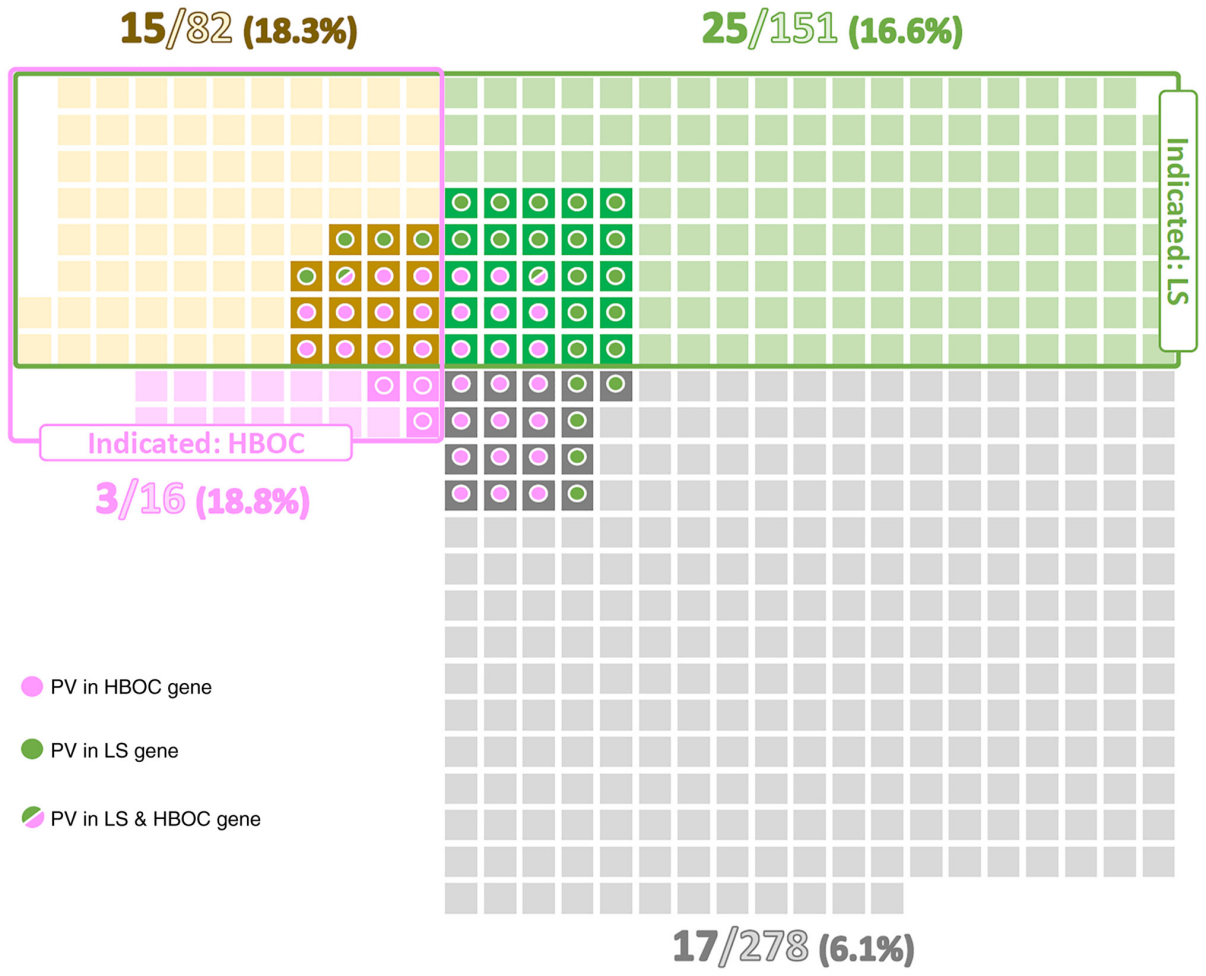
Distribution of PV carriers in patient subgroups based on criteria for germline genetic testing for LS and HBOC. Squares colored in green, pink, yellow and grey represent individual patients fulfilling criteria for LS only, criteria for HBOC only, both criteria or not fulfilling any criteria, respectively. Circles denote carriers of PV in LS genes (green), HBOC genes (pink) or both (green/pink). HBOC, hereditary breast and ovarian cancer; LS, Lynch syndrome; PV, pathogenic variant.

Among EC patients indicated for germline genetic testing according to the above-mentioned criteria, the proportions of PV carriers fulfilling criteria for LS, HBOC, and both conditions were similar (16.6, 18.8, and 18.3%, respectively). These proportions were approximately three-times higher than in EC patients not fulfilling any criteria for germline genetic testing (6.1%; Fig. 1). As expected, the highest proportion of PV in LS genes (11.3%) was detected in a subgroup of patients fulfilling criteria only for LS testing. Similarly, patients meeting solely the HBOC testing criteria had the highest frequency (18.8%) of PV in HBOC genes. Even though the overall percentage of PV carriers differed between subgroups of patients meeting both LS + HBOC genetic testing criteria (18.3%) and not fulfilling any criteria (6.1%), the ratio of carriers of PV in LS:HBOC genes in these two subgroups was similar (5:11 vs. 5:12; Table II, Fig. 1). On the other hand, highly penetrant genes (*MLH1, MSH2, BRCA1*) were predominantly affected in the subgroup fulfilling both criteria, whereas the subgroup of non-indicated patients was characterized by PV in less penetrant genes (*MHS6, ATM*).

Moreover, among non-indicated patients we found 2 PVs in HBOC genes in subset of 41 patients with double primary EC and breast cancer (BC; 1×*ATM*, 1×*BRCA1*; 2/41; 4.9%) and 3 PVs in HBOC genes in subset of 31 patients with EC and BC in family cancer history (2×*BRCA2*, 1×*CHEK2*; 3/31; 9.7%).

### Germline PV in other candidate cancer predisposition genes

The overall prevalence of PV in remaining candidate genes (identified in 48 out of 207 genes) was significantly higher in EC patients (66/527; 12.5%) compared to controls (139/1662; 8.4%; P=0.004; Table SIV). Eight EC patients (and no PMC) carried a coincidental PV in EC-predisposition and candidate genes. Excluding all 60 carriers of PV in EC-predisposition genes, the frequency of PV carriers in other candidate genes was still significantly higher in 467 EC patients (N=58; 12.4%) in comparison to 1616 PMC (N=139; 8.6%; P=0.01). The most frequent PV were found in *MUTYH* (monoallelic PV in 5/467, 1.1%) and *FANCA* (4/467; 0.8%). Their frequencies, however, did not differ from that in PMC (*MUTYH*−18/1616, 1.1%; *FANCA*−10/1616, 0.6%).

Interestingly, three patients carried germline truncating variant in the genes coding for DNA polymerases (two in *POLE* and one in *POLD1*) that have been linked to EC-predisposition previously (5). In contrast, only one *POLE* and no *POLD1* mutation was detected in PMC. Thus, the overall frequency of PV in DNA polymerases was significantly higher in EC-predisposition gene negative patients (3/467; 0.6%) than in PMC (1/1616; 0.06%; OR=10.44; 95% CI 1.08-100.51; P=0.012).

Regarding subgroups of patients based on indication criteria for genetic testing, the frequency of PV in candidate predisposition genes (after excluding the carriers of PV in EC-predisposition genes) was significantly higher in patients fulfilling both indication criteria for LS + HBOC (14/67; 20.9%) in comparison to subgroup of patients fulfilling no genetic testing criteria (28/261; 10.7%; P=0.04, Table SIV). The frequencies of PV in patients meeting indication criteria for LS only and HBOC only did not differ significantly (14/126; 11.1 and 2/13; 15.4%, respectively).

### Clinicopathological characteristics in germline PV carriers

**Figure 2.**
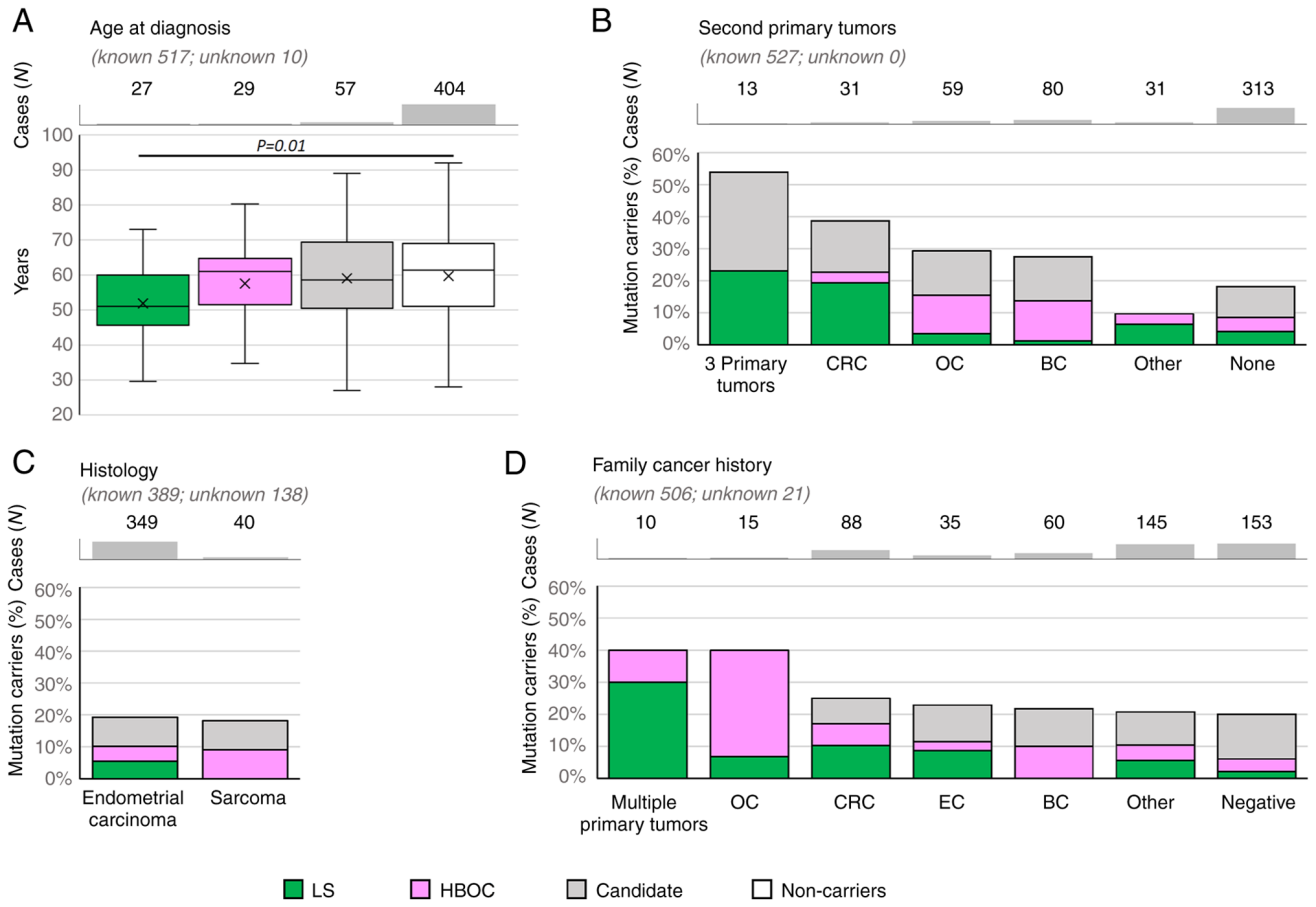
Relative proportion of mutation carriers in clinicopathological subgroups, including (A) age at diagnosis, (B) second primary tumors, (C) histology and (D) family cancer history in 527 patients. Error bars in (A) indicate the first and the fourth quartile. BC, breast cancer; CRC, colorectal cancer; EC, endometrial cancer; HBOC, hereditary breast and ovarian cancer; LS, Lynch syndrome; OC, ovarian cancer.

The median age at EC onset was significantly lower only in patients with PV in LS genes compared to non-carriers (51.0 vs. 61.4 years, P=0.01, Fig. 2A).

Concerning the histology subtypes (Fig. 2C), the overall frequency of PV in EC-predisposition genes was similar in patients with endometrial carcinoma to those with sarcoma subtypes (39/349, 11.2% and 4/40, 10.0%; respectively); however, no carrier of PV in LS gene was diagnosed with sarcoma. Interestingly, two out of eight patients diagnosed with precancerous EIN (endometrial intraepithelial neoplasia) carried a PV in *BRCA1*. Unfortunately, the histologic subtypes of endometrial carcinomas other than endometrioid were rarely represented, thus the frequencies of PVs in these subgroups cannot be calculated and compared.

Analysis of patients with second primary tumors (Fig. 2B) revealed that the highest frequency of PV in EC-predisposition genes was found in patients with 3 primary tumors and in patients with second primary colorectal cancer (CRC). The proportion between carriers of PV in LS and HBOC genes respected the corresponding indication criteria: the carriers of LS gene variants were enriched in patients with EC + CRC and 3 primary tumors. Accordingly, all 13 patients with 3 primary tumors developed either CRC (N=5) and/or ovarian cancer (OC; N=10). The carriers of PV in HBOC genes were more frequent in patients with EC + OC and EC + BC.

When considering family cancer history (Fig. 2D), the highest frequency (reaching 40%) of PV in EC-predisposition genes were found in small subgroups of patients with family history of multiple primary tumors and family history of ovarian tumors. Not surprisingly, predominant tumor types in a family were in concordance with the elevated frequencies of PV in LS or HBOC genes.

The prevalence of carriers of PV in candidate predisposition genes did not differ from that of non-carriers in any of the clinicopathological categories.

The information about immunohistochemistry and microsatellite instability in EC tumor specimens was unavailable.

## Discussion

**Figure 3.**
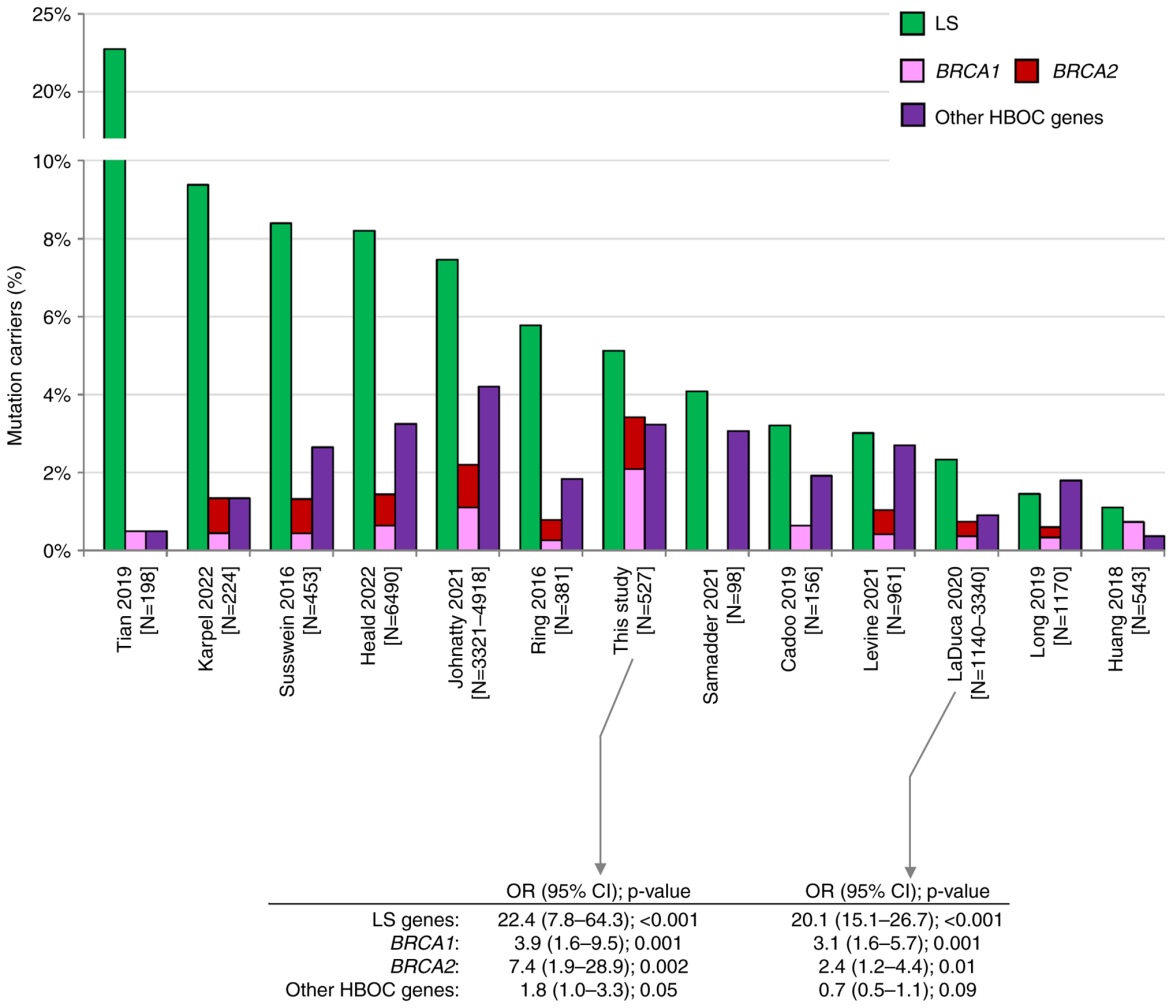
Comparison among previously published studies describing germline PV in patients with endometrial cancer (6–15,28,29). Green, pink, red and purple bars represent the prevalence of PV in LS genes, *BRCA1, BRCA2* and other HBOC genes (*ATM, BARD1, BRIP1, CDH1, CHEK2, NF1, PALB2, PTEN, RAD51C, RAD51D, STK11* and *TP53*), respectively. CI, confidence interval; HBOC, hereditary breast and ovarian cancer; LS, Lynch syndrome; N, number; OR, odds ratio; PV, pathogenic variant.

Pathogenic germline alterations in LS genes are considered the most significant genetic risk factor for EC predisposition (5). In our study, the carriers of PV in LS genes represented 5.1% of all analyzed EC patients. This frequency is approximately in the middle of frequencies reported by other studies (Fig. 3). Variable frequencies result from inconsistent patients' enrollment criteria. Studies reporting the highest frequency (Tian *et al* (7), Karpel *et al* (13), Susswein *et al* (14), Heald *et al* (15) with 22.7, 9.4, 8.4, and 8.2% of LS PV carriers, respectively) analyzed high-risk EC patients enriched in individuals with familial LS criteria or in patients with positive MMR gene immunohistochemistry [Tian *et al* (7)]. In contrast, the lowest frequency of PV in LS genes was reported by studies with unselected EC cases, including Huang *et al* (28) (1.1%), a study of EC samples from The Cancer Genome Atlas (TCGA). We have found similar differences as we identified 22/233 (9.4%) vs. 5/294 (1.7%) carriers of PV in LS genes in LS-indicated vs. LS non-indicated patients, respectively (Fig. 1). Interestingly, despite differences in frequencies of PV in EC patients, the risk of EC development in LS PV carriers was similar in our and LaDuca *et al* (29) study (OR 22.4 and 20.1, respectively; Fig. 3), the only study among those previously published that quantified the EC risk associated with PV in LS genes.

Even though only less than 20% of analyzed EC patients (98/527, 18.6%) met the HBOC germline genetic testing criteria, the overall frequencies of PV carriers in *BRCA1/BRCA2* were unusually high in contrast to other studies (Fig. 3). We identified 11 PVs in *BRCA1* (2.1%) and 7 PVs in *BRCA2* (1.3%). Compared to frequencies of PVs in controls we calculated the risks OR=3.9 for *BRCA1* and OR=7.4 for *BRCA2* (Table II). The risk of EC development associated with *BRCA1* and *BRCA2* mutations was substantially lower than in LS carriers, and similar to EC risk reported previously by LaDuca *et al* (29). Our results suggest that PV in *BRCA1*/*BRCA2* are associated with at least moderate EC risk. Among 16 EC patients meeting only the HBOC criteria, three harbored *BRCA1*/*BRCA2* mutation. This was also documented by results of a small study by Vietri *et al* (30), who identified PV in *BRCA1*/*BRCA2* in 9/21 hereditary EC patients fulfilling HBOC testing criteria. In the group of 82 patients meeting both LS and HBOC testing criteria, *BRCA1* PV were more frequent than PV in LS genes. Moreover, up to 5 and 10% of PVs in HBOC genes were identified in non-indicated EC patients with BC in personal or family cancer history, respectively. This further implies that the diagnosis of EC should be considered as a part of indication criteria for HBOC germline genetic testing irrespective to EC histology subtype. Among PV carriers in other HBOC genes, PV in *CHEK2* and *ATM* were the most frequent. Importantly, PV in *CHEK2* were associated with moderately increased risk (OR=3.2, P=0.04). Mutations in *CHEK2* were associated with predisposition to EC in several studies previously (31).

Our analysis of other candidate genes showed that only PVs in *POLD1* and *POLE* (three truncating variants, one in *POLD1*, two in *POLE*) were significantly associated with EC risk. Germline truncating variants in DNA polymerase genes in our EC patients conferred about 10-times increased risk of EC development. Germline missense PV in both DNA polymerase genes affecting exonuclease domains were previously linked to EC predisposition (5) and their specific somatic missense PV represent important predictive markers for favorable prognosis and/or immune checkpoint therapy in EC patients (32–34). However, the exact risk as well as the overall role of germline truncating variants needs to be further validated in larger cohorts due to the low frequency of *POLD1* and *POLE* mutation carriers.

Analysis of clinicopathological characteristics confirmed an earlier age at disease onset in carriers of LS gene mutations in comparison to non-carriers as referred in other studies (6,7,9,10). The age at EC onset varied even among the carriers of PV in particular LS genes: the carriers of PV in *MSH6* had later age at onset (56 years) compared to the *MLH1*/*MSH2* PV carriers (48 years), as previously described by Tian *et al* (7). Interestingly, the age at EC onset in carriers of PV in HBOC genes did not differ from non-carriers.

As expected, other differences in clinicopathological characteristics largely corresponded to subgroups of patients classified according to the germline genetic testing criteria. PV in LS genes were most frequently identified in patients with ≥3 primary tumors or second primary CRC in personal cancer history, or multiple primary tumors/CRC in family cancer history. Similarly, carriers of PV in HBOC genes recruited in majority from individuals with BC/OC in personal or family cancer history. On the other hand, clinicopathological characteristics did not differ in carriers of candidate EC-predisposition genes and non-carriers.

Generally and as expected, we have identified the majority of PV in the groups of patients fulfilling genetic testing criteria for LS or HBOC with majority of PV in genes related to a corresponding cancer syndrome. Overall, 43/60 PV (71.7%) carriers were indicated for germline genetic testing. Importantly, remaining 17 PV carriers, who would not be indicated for genetic testing using current indication criteria, still represent a significant proportion (28.3%) of cases carrying a germline PV in the LS (*MLH1, MSH6*) or the HBOC (*ATM, BRCA1, BRCA2, BRIP1, CHEK2*) genes. Of these, two had double primary tumors and an additional 10 had a positive family cancer history. The frequency of PV carriers among EC patients with double primary tumors was 15.4% (33/214). While we have found eight PV carriers in HBOC genes and four carriers in LS genes (including a patient with co-occurrence of *BRCA1* and *MLH1* PV and diagnosed with EC, OC, and BC) in the group of 69 patients with EC and OC (11.6%), we have identified eight carriers of LS genes mutation and only one additional carrier of the *CHEK2* gene mutation (a patient with EC, CRC, and melanoma) in the group of 34 patients with EC and CRC (26.5%). This suggests that the presence of double primary tumors could potentially represent a sole indication criterion for germline genetic testing, as indicated by previous studies (19,20,35,36).

Strengths of our study include homogeneity of studied population consisting of Caucasians, Slavs of the Czech origin and inclusion of PMC that allowed calculation of overall/gene-level risks for EC development. Study limitations include retrospective design and unavailability of EC tumors immunohistochemistry, microsatellite instability and mutation status of *POLE*, which prevented us from correlating presence of germline mutations with different molecular subtypes of EC. Moreover, as approximately half of the analyzed EC patients (292/527, 55.4%) were recruited from the CZECANCA consortium (focused on analyses of genetic cancer predisposition), we cannot exclude a potential bias toward enriched prevalence of PV carriers. To minimize this bias, we divided all enrolled patients according to the testing criteria and analyzed them independently.

In conclusion, over 11% of EC patients carried a germline PV in genes associated with established germline cancer predisposition. EC patients fulfilling LS criteria had five-times higher chance to carry a LS gene PV than EC patients not fulfilling criteria for germline genetic testing. Presence of PV in LS gene increases the EC risk 20-fold when compared with non-carriers. However, 28.3% of PV carriers in clinically relevant genes would not be indicated for germline genetic testing using current indication criteria. Therefore, we believe that EC as a second primary tumor in proband or occurrence of EC in a family cancer history should be considered within the indication criteria for germline genetic testing. This is of particular importance for countries where reflex testing is not routinely performed in EC patients.

## Supplementary Material

Supporting Data

## Figures and Tables

**Table I.** Clinicopathological characteristics of 527 patients with EC.

Variables	All patients with EC (N=527)	LS only (N=151)	HBOC only (N=16)	LS + HBOC (N=82)	Non-indicated (N=278)
Age at EC diagnosis					
Mean, years	59.1	50.8	59.0	51.3	65.8
Median, years	60.5	47.8	57.0	49.0	65.3
Range, years	24-92	24-91	51-73	29-82	50-92
<50 years, n (%)	120 (23.2)	79 (53.4)	0	41 (51.3)	0
≥50 years, n (%)	397 (76.8)	69 (46.6)	15 (100.0)	39 (48.8)	274 (100.0)
N.A., n	10	3	1	2	4
Histology of uterine malignances, n (%)					
Endometrial carcinoma	349 (89.7)	76 (85.4)	8 (72.7)	48 (100.0)	217 (90.0)
Endometrioid adenocarcinoma	284 (73.0)	65 (73.0)	7 (63.6)	44 (91.7)	168 (69.7)
Serous	35 (9.0)	4 (4.5)	1 (9.1)	3 (6.3)	27 (11.2)
Clear cell	7 (1.8)	2 (2.2)	0	0	5 (2.1)
Undifferentiated	3 (0.8)	0	0	0	3 (1.2)
Mixed (endometroid/serous)	3 (0.8)	0	0	0	3 (1.2)
Mixed (endometroid/serous/clear cell)	1 (0.3)	1 (1.1)	0	0	0
Mixed (endometroid/clear cell)	4 (1.0)	3 (3.4)	0	0	1 (0.4)
EIN	8 (2.1)	1 (1.1)	0	1 (2.1)	6 (2.5)
Unspecified	4 (1.0)	0	0	0	4 (1.7)
Sarcoma	40 (10.3)	13 (14.6)	3 (27.3)	0	24 (10.0)
Leiomyosarcoma	32 (8.2)	9 (10.1)	2 (18.2)	0	21 (8.7)
Undifferentiated	2 (0.5)	0	0	0	2 (0.8)
Endometrial stromal sarcoma	3 (0.8)	2 (2.2)	0	0	1 (0.4)
Unspecified	3 (0.8)	2 (2.2)	1 (9.1)	0	0
Unknown malignant tumor of corpus uteri	138	62	5	34	37
FIGO grade, n (%)					
1	123 (35.9)	35 (48.6)	4 (40.0)	16 (45.7)	68 (30.1)
2	100 (29.2)	15 (20.8)	3 (30.0)	12 (34.3)	70 (31.0)
3	120 (35.0)	22 (30.6)	3 (30.0)	7 (20.0)	88 (38.9)
N.A.	184	79	6	47	52
FIGO stage, n (%)					
0	8 (2.8)	1 (2.1)	0	1 (4.2)	6 (2.8)
I	176 (60.9)	33 (68.8)	4 (66.7)	17 (70.8)	122 (57.8)
II	38 (13.1)	5 (10.4)	1 (16.7)	2 (8.3)	30 (14.2)
III	48 (16.6)	8 (16.7)	1 (16.7)	2 (8.3)	37 (17.5)
IV	19 (6.6)	1 (2.1)	0	2 (8.3)	16 (7.6)
N.A.	238	103	10	58	67
Multiple primary tumors in personal history, n (%)					
Present	214 (40.6)	69 (45.7)	16 (100.0)	82 (100.0)	47 (16.9)
Absent	313 (59.4)	82 (54.3)	0	0	231 (83.1)
Multiple primary tumors in personal history, n (%)					
CRC	31 (5.9)	31 (20.5)	0	0	0
OC	59 (11.2)	0	1 (6.3)	58 (70.7)	0
BC	80 (15.2)	14 (9.3)	15 (93.8)	13 (15.9)	38 (13.7)
Triple primary EC+(BC/OC/CRC)	13 (2.5)	2 (1.3)	0	11 (13.4)	0
Other	31 (5.9)	22 (14.6)	0	0	9 (3.2)
None	313 (59.4)	82 (54.3)	0	0	231 (83.1)
Family cancer history (first/second degree), n (%)					
Positive	353 (69.8)	120 (81.6)	13 (100.0)	56 (73.7)	164 (60.7)
Negative	153 (30.2)	27 (18.4)	0	20 (26.3)	106 (39.3)
Unknown	21	4	3	6	8
Tumors in family history, n (%)					
EC	35 (6.9)	14 (9.5)	1 (7.7)	6 (7.9)	14 (5.2)
CRC	88 (17.4)	39 (26.5)	4 (30.8)	15 (19.7)	30 (11.1)
OC	15 (3.0)	7 (4.8)	1 (7.7)	5 (6.6)	2 (0.7)
BC	60 (11.9)	14 (9.5)	3 (23.1)	9 (11.8)	34 (12.6)
Multiple (EC/OC/CRC)	10 (2.0)	10 (6.8)	0	0	0
Other	145 (28.7)	36 (24.5)	4 (30.8)	21 (27.6)	84 (31.1)
None	153 (30.2)	27 (18.4)	0	20 (26.3)	106 (39.3)
Unknown	21	4	3	6	8

Percentages were calculated from the overall number of patients with known characteristics. BC, breast cancer; CRC, colorectal cancer; EC, endometrial cancer; EIN, endometrial intraepithelial neoplasia; FIGO, The International Federation of Gynecology and Obstetrics; HBOC, hereditary breast and ovarian cancer; LS, Lynch syndrome; N, number; N.A., not available; OC, ovarian cancer.

**Table II.** Frequencies of germline PV in 19 ec-predisposition genes.

		Indication for germline genetic testing						All patients with EC vs. PMC	
				
Gene group	Germline PV	LS, n (%) (N=151)	HBOC, n (%) (N=16)	LS+HBOC, n (%) (N=82)	Non-indicated, n (%) (N=278)	All patients with EC, n (%) (N=527)	PMC, n (%) (N=1662)		
								OR (95% CI)	P-value
LS	*MLH1^a^*	3 (2.0)	0	2^a^ (2.4)	1 (0.4)	6^a^ (1.1)	1 (0.1)	19.1	1.3×10^−4^
								(2.3-159.1)	
	*MSH2^b^*	6^b^ (4.0)	0	2 (2.4)	0	8^b^ (1.5)	0	N.A.	
	*MSH6*	8 (5.3)	0	1 (1.2)	4 (1.4)	13 (2.4)	0	N.A.	
	*PMS2*	0	0	0	0	0	3 (0.2)	N.A.	
	*EPCAM*	0	0	0	0	0	0	N.A.	
	All LS	17	0	5 (6.1)	5 (1.8)	27 (5.1)	4 (0.2)	22.4	1.8×10^−17^
	genes	(11.3)						(7.8-64.3)	
HBOC	*ATM^b^*	1^b^ (0.7)	0	1 (1.2)	3 (1.1)	5^b^ (1.0)	7 (0.4)	2.3	0.2
								(0.2-7.2)	
	*BARD1*	0	0	1 (1.2)	0	1 (0.2)	0	N.A.	
	*BRCA1^a^*	2 (1.3)	2 (12.5)	6^a^ (7.3)	1 (0.4)	11^a^ (2.1)	9 (0.5)	3.9	1.0×10^−3^
								(1.6-9.5)	
	*BRCA2*	1 (0.7)	1 (6.3)	0	5 (1.8)	7 (1.3)	3 (0.2)	7.4	2.0×10^−3^
								(1.9-28.9)	
	*BRIP1*	0	0	0	1 (0.4)	1 (0.2)	3 (0.2)	1.1	>0.9
								(0.1-10.1)	
	*CDH1*	0	0	0	0	0	0	N.A.	
	*CHEK2*	3 (2.0)	0	1 (1.2)	2 (0.7)	6 (1.1)	6 (0.4)	3.2	4.0×10^−2^
								(1.0-9.9)	
	*NF1*	0	0	0	0	0	1 (0.1)	N.A.	
	*PALB2*	1 (0.7)	0	0	0	1 (0.2)	8 (0.5)	0.4	0.4
								(0.1-3.1)	
	*PTEN*	1 (0.7)	0	0	0	1 (0.2)	1 (0.1)	3.2	0.4
								(0.2-50.5)	
	*RAD51C*	0	0	2 (2.4)	0	2 (0.4)	2 (0.1)	3.2	0.2
								(0.4-22.5)	
	*RAD51D*	0	0	0	0	0	0	N.A.	
	*STK11*	0	0	0	0	0	0	N.A.	
	*TP53*	0	0	0	0	0	2 (0.1)	N.A.	
	All	9 (6.0)	3 (18.8)	11 (13.4)	12 (4.3)	35 (6.6)	42 (2.5)	2.7	7.9×10^−5^
	HBOC							(1.7-4.3)	
All genes	All PV	26	3	16	17	62	46		
All genes	All	25^b^	3 (18.8)	15^a^ (18.3)	17 (6.1)	60^a,b^ (11.4)	46 (2.8)		
	carriers	(16.6)							

a Double PV carrier in *MLH1/BRCA1*.

b Double PV carrier in *MSH2/ATM*. Frequencies of germline PV found in a subgroup of patients fulfilling criteria for germline genetic testing for LS, HBOC, LS + HBOC, individuals not fulfilling any criteria (non-indicated), an aggregated group of all EC patients, and a group of PMC, respectively. CI, confidence interval; EC, endometrial cancer; HBOC, hereditary breast and ovarian cancer; LS, Lynch syndrome; N, number; N.A., not available; OR, odds ratio; PMC, population-matched controls; PV, pathogenic variant.

## Data Availability

The datasets generated and/or analyzed during the current study are not publicly available due to restrictions imposed by national regulatory authorities but are available from the corresponding author on reasonable request.

## Abbreviations

ACMG: American College of Medical Genetics
BC: breast cancer
CI: confidence interval
CNV: copy number variation
CRC: colorectal cancer
EC: endometrial cancer
EIN: endometrial intraepithelial neoplasia
FIGO: The International Federation of Gynecology and Obstetrics
GGT: germline genetic testing
HBOC: hereditary breast and ovarian cancer
LS: Lynch syndrome
MMR: mismatch repair
N: number
N.A.: not available
NCCN: National Comprehensive Cancer Network
NGS: next generation sequencing
OC: ovarian cancer
OR: odds ratio
P/LP: pathogenic/likely pathogenic
PMC: population-matched controls
PV: pathogenic variant
VUS: variant of uncertain significance
