# Supplementary material for: Germline multigene panel testing of patients with endometrial cancer
Source: Oncol Lett. 2023 Apr 12;25(6):216. doi: 10.3892/ol.2023.13802 (PMC10157349; doi:10.3892/ol.2023.13802)
Supplement: Supporting Data [file Supplementary_Data.pdf]

Table SI. List of all 10 patient sample contributors participating in the study.

Patient sample contributors	Number of patients included in the study
The Bank of Clinical Samples, First Faculty of Medicine, Charles University, Prague, Czech Republic	235
Institute of Medical Biochemistry and Laboratory Diagnostics, First Faculty of Medicine, Charles University and General University Hospital in Prague, Prague, Czech Republic	51
Department of Cancer Epidemiology and Genetics, Masaryk Memorial Cancer Institute, Brno, Czech Republic	29
Department of Medical Genetics, AGEL Laboratories, AGEL Research and Training Institute, Novy Jicin, Czech Republic	15
Centre for Medical Genetics and Reproductive Medicine, Gennet, Prague, Czech Republic	140
Institute of Biology and Medical Genetics, First Faculty of Medicine and General University Hospital in Prague, Prague, Czech Republic	25
Department of Medical Genetics, GHC Genetics, Prague, Czech Republic	4
Department of Medical Genetics, Faculty of Medicine in Pilsen, Charles University, and University Hospital Pilsen, Pilsen, Czech Republic	10
Department of Medical Genetics, Pronatal, Prague, Czech Republic	2
Department of Medical Genetics, University Hospital Olomouc, Faculty of Medicine and Dentistry, Palacky University Olomouc, Czech Republic	16

Table SII. List of all 226 genes included in the CZECANCA panel with used transcript variants and full names.

A, Established EC-predisposition genes (N=19)		
Gene name	Transcript variant	Gene name (alternative denominations)
ATM	NM_000051.3	Ataxia telangiectasia mutated
BARD1	NM_000465.3	BRCA1 associated RING domain 1
BRCA1	NM_007294.3	Breast cancer 1, early onset
BRCA2	NM_000059.3	Breast cancer 2, early onset
BRIP1	NM_032043.2	BRCA1 interacting protein C-terminal helicase 1
CDH1	NM_004360.4	Cadherin 1, type 1, E-cadherin
EPCAM[a]	NM_002354.2	Epithelial cell adhesion molecule
CHEK2	NM_007194.3	Checkpoint kinase 2
MLH1	NM_000249.3	mutL homolog 1, colon cancer, nonpolyposis type 2
MSH2	NM_000251.2	mutS homolog 2, colon cancer, nonpolyposis type 1
MSH6	NM_000179.2	mutS homolog 6
NF1	NM_000267.3	Neurofibromin 1
PALB2	NM_024675.3	Partner and localizer of BRCA2
PMS2	NM_000535.6	PMS1 homolog 2
PTEN	NM_000314.6	Phosphatase and tensin homolog
RAD51C	NM_058216.2	RAD51 homolog C
RAD51D	NM_002878.3	RAD51 homolog D
STK11	NM_000455.4	Serine/threonine kinase 11
TP53	NM_000546.5	Tumor protein p53
B, Candidate genes (N=207)		
Gene name	Transcript variant	Gene name (alternative denominations)

AIP	NM_003977.3	Aryl hydrocarbon receptor interacting protein
ALK	NM_004304.4	Anaplastic lymphoma kinase
APC	NM_000038.5	Adenomatous polyposis coli
APEX1	NM_001641.3	APEX nuclease (multifunctional DNA repair enzyme) 1
ATMIN	NM_015251.2	ATM interactor
ATR	NM_001184.3	Ataxia telangiectasia and Rad3 related
ATRIP	NM_130384.2	ATR interacting protein
AURKA	NM_198433.2	Aurora kinase A
AXIN1	NM_003502.3	Axin 1
BABAM1	NM_001033549.2	BRISC and BRCA1 A complex member 1
BAP1	NM_004656.3	BRCA1 associated protein-1 (ubiquitin carboxy-terminal hydrolase)
BLM	NM_000057.3	Bloom syndrome, RecQ helicase-like
BMPR1A	NM_004329.2	Bone morphogenetic protein receptor, type IA
BRAP	NM_006768.4	BRCA1 associated protein
BRCC3	NM_024332.3	BRCA1/BRCA2-containing complex, subunit 3
BRE	NM_004899.4	Brain and reproductive organ-expressed (TNFRSF1A modulator)
BUB1B	NM_001211.5	Budding uninhibited by benzimidazoles 1 homolog beta
CASP8	NM_001080125.1	Caspase 8, apoptosis-related cysteine peptidase
CCND1	NM_053056.2	Cyclin D1
CDC73	NM_024529.4	Cell division cycle 73, Paf1/RNA polymerase II complex component, homolog

CDK4	NM_000075.3	Cyclin-dependent kinase 4
CDKN1B	NM_004064.4	Cyclin-dependent kinase inhibitor 1B (p27, Kip1)
CDKN1C	NM_000076.2	Cyclin-dependent kinase inhibitor 1C (p57, Kip2)
CDKN2A	NM_000077.4	Cyclin-dependent kinase inhibitor 2A
CEBPA	NM_004364.4	CCAAT/enhancer binding protein (C/EBP), alpha
CEP57	NM_014679.4	Centrosomal protein 57kDa
CLSPN	NM_022111.3	Claspin
CSNK1D	NM_001893.4	Casein kinase 1, delta
CSNK1E	NM_001894.4	Casein kinase 1, epsilon
CWF19L2	NM_152434.2	CWF19-like 2, cell cycle control
CYLD	NM_015247.2	Cylindromatosis (turban tumor syndrome)
DCLRE1C	NM_001033855.2	DNA cross-link repair 1C
DDB2	NM_000107.2	Damage-specific DNA binding protein 2, 48kDa
DHFR	NM_000791.3	Dihydrofolate reductase
DICER1	NM_030621.4	Dicer 1, ribonuclease type III
DIS3L2	NM_152383.4	DIS3 like 3'-5' exoribonuclease 2
DMBT1	NM_007329.2	Deleted in malignant brain tumors 1
DMC1	NM_007068.3	DMC1 dosage suppressor of mck1 homolog, meiosis-specific homologous recombination
DNAJC21	NM_194283.3	DnaJ (Hsp40) homolog, subfamily C, member 21
DPYD	NM_000110.3	Dihydropyrimidine dehydrogenase
EGFR	NM_005228.3	Epidermal growth factor receptor
EMSY	NM_001300942.1	EMSY transcriptional repressor, BRCA2 interacting
EPHX1	NM_000120.3	Epoxide hydrolase 1

ERCC1	NM_001983.3	Excision repair cross-complementing rodent repair deficiency, complementation group 1
ERCC2	NM_000400.3	Excision repair cross-complementing rodent repair deficiency, complementation group 2
ERCC3	NM_000122.1	Excision repair cross-complementing rodent repair deficiency, complementation group 3
ERCC4	NM_005236.2	Excision repair cross-complementing rodent repair deficiency, complementation group 4
ERCC5	NM_000123.3	Excision repair cross-complementing rodent repair deficiency, complementation group 5
ERCC6	NM_000124.3	Excision repair cross-complementing rodent repair deficiency, complementation group 6
ESR1	NM_000125.3	Estrogen receptor 1
ESR2	NM_001437.2	Estrogen receptor 2 (ER beta)
EXO1	NM_006027.4	Exonuclease 1
EXT1	NM_000127.2	Exostosin 1
EXT2	NM_000401.3	Exostosin 2
EYA2	NM_005244.4	Eyes absent homolog 2
EZH2	NM_004456.4	Enhancer of zeste homolog 2
FAAP24	NM_152266.4	FA core complex associated protein 24
FAM175A	NM_139076.2	Family with sequence similarity 175, member A
FAM175B	NM_032182.3	Family with sequence similarity 175, member B
FAN1	NM_014967.4	FANCD2/FANCI-associated nuclease 1

FANCA	NM_000135.2	Fanconi anemia, complementation group A
FANCB	NM_001018113.2	Fanconi anemia, complementation group B
FANCC	NM_000136.2	Fanconi anemia, complementation group C
FANCD2	NM_033084.4	Fanconi anemia, complementation group D2
FANCE	NM_021922.2	Fanconi anemia, complementation group E
FANCF	NM_022725.3	Fanconi anemia, complementation group F
FANCG	NM_004629.1	Fanconi anemia, complementation group G
FANCI	NM_001113378.1	Fanconi anemia, complementation group I
FANCL	NM_001114636.1	Fanconi anemia, complementation group L
FANCM	NM_020937.3	Fanconi anemia, complementation group M
FBXW7	NM_033632.3	F-box and WD repeat domain containing 7, E3 ubiquitin protein ligase
FH	NM_000143.3	Fumarate hydratase
FLCN	NM_144997.5	Folliculin
GADD45A	NM_001924.3	Growth arrest and DNA-damage-inducible, alpha
GATA2	NM_001145661.1	GATA binding protein 2
GPC3	NM_004484.3	Glypican 3
GRB7	NM_001242442.1	Growth factor receptor-bound protein 7
HELQ	NM_133636.3	Helicase, POLQ-like
HNF1A	NM_001306179.1	HNF1 homeobox A

HOXB13	NM_006361.5	Homeobox B13
HRAS	NM_005343.3	v-Ha-ras Harvey rat sarcoma viral oncogene homolog
HUS1	NM_004507.3	HUS1 checkpoint homolog
CHEK1	NM_001114122.2	Checkpoint kinase 1
KAT5	NM_182710.2	K(lysine) acetyltransferase 5
KCNJ5	NM_000890.3	Potassium inwardly-rectifying channel, subfamily J, member 5
KIT	NM_000222.2	V-kit Hardy-Zuckerman 4 feline sarcoma viral oncogene homolog
LIG1	NM_000234.2	Ligase I, DNA, ATP-dependent
LIG3	NM_013975.3	Ligase III, DNA, ATP-dependent
LIG4	NM_002312.3	Ligase IV, DNA, ATP-dependent
LMO1	NM_002315.2	LIM domain only 1
LRIG1	NM_015541.2	Leucine-rich repeats and immunoglobulin-like domains 1
MAX	NM_002382.4	MYC associated factor X
MCPH1	NM_024596.4	Microcephalin 1
MDC1	NM_014641.2	Mediator of DNA-damage checkpoint 1
MDM2	NM_002392.5	Mdm2, p53 E3 ubiquitin protein ligase homolog
MDM4	NM_002393.4	Mdm4 p53 binding protein homolog
MEN1	NM_000244.3	Multiple endocrine neoplasia I
MET	NM_001127500.2	Met proto-oncogene (hepatocyte growth factor receptor)
MGMT	NM_002412.4	O-6-methylguanine-DNA methyltransferase
MLH3	NM_001040108.1	mutL homolog 3
MMP8	NM_002424.2	Matrix metallopeptidase 8 (neutrophil collagenase)
MPL	NM_005373.2	Myeloproliferative leukemia virus oncogene

MRE11	NM_005591.3	MRE11 meiotic recombination 11 homolog A
MSH3	NM_002439.4	mutS homolog 3
MSH5	NM_002441.4	mutS homolog 5
MSR1	NM_138715.2	Macrophage scavenger receptor 1
MUS81	NM_025128.4	MUS81 endonuclease homolog
MUTYH	NM_001128425.1	mutY homolog
NAT1	NM_000662.7	N-acetyltransferase 1 (arylamine N-acetyltransferase)
NBN	NM_002485.4	Nibrin
NCAM1	NM_001242607.1	Neural cell adhesion molecule 1
NELFB	NM_015456.4	Cofactor of BRCA1
NF2	NM_000268.3	Neurofibromin 2 (merlin)
NFKBIZ	NM_031419.3	Nuclear factor of kappa light polypeptide gene enhancer in B-cells inhibitor, zeta
NHEJ1	NM_024782.2	Nonhomologous end-joining factor 1
NSD1	NM_022455.4	Nuclear receptor binding SET domain protein 1
OGG1	NM_002542.5	8-oxoguanine DNA glycosylase
PARP1	NM_001618.3	Poly (ADP-ribose) polymerase 1
PCNA	NM_002592.2	Proliferating cell nuclear antigen
PHB	NM_002634.3	Prohibitin
PHOX2B	NM_003924.3	Paired-like homeobox 2b
PIK3CG	NM_002649.3	Phosphatidylinositol-4,5-bisphosphate 3-kinase, catalytic subunit gamma
PLA2G2A	NM_000300.3	Phospholipase A2, group IIA
PMS1	NM_000534.4	PMS1 postmeiotic segregation increased 1
POLB	NM_002690.2	Polymerase (DNA directed), beta
POLD1	NM_002691.3	Polymerase (DNA directed), delta 1, catalytic subunit

POLE	NM_006231.3	Polymerase (DNA directed), epsilon, catalytic subunit
PPM1D	NM_003620.3	Protein phosphatase, Mg2+/Mn2+ dependent, 1D
PREX2	NM_024870.3	Phosphatidylinositol-3,4,5-trisphosphate-dependent Rac exchange factor 2
PRF1	NM_001083116.1	Perforin 1 (pore forming protein)
PRKAR1A	NM_212471.2	Protein kinase, cAMP-dependent, regulatory, type I, alpha
PRKDC	NM_006904.6	Protein kinase, DNA-activated, catalytic polypeptide
PTCH1	NM_000264.3	Patched 1
PTTG2	NM_006607.2	Pituitary tumor-transforming 2
RAD1	NM_002853.3	RAD1 homolog
RAD17	NM_133338.2	RAD17 homolog
RAD18	NM_020165.3	RAD18 homolog
RAD23B	NM_002874.4	RAD23 homolog B
RAD50	NM_005732.3	RAD50 homolog
RAD51	NM_133487.3	RAD51 homolog
RAD51AP1	NM_001130862.1	RAD51 associated protein 1
RAD51B	NM_133509.3	RAD51 homolog B
RAD52	NM_134424.3	RAD52 homolog
RAD54B	NM_012415.3	RAD54 homolog B
RAD54L	NM_003579.3	RAD54-like
RAD9A	NM_004584.2	RAD9 homolog A
RB1	NM_000321.2	Retinoblastoma 1
RBBP8	NM_002894.2	Retinoblastoma binding protein 8
RECQL	NM_002907.3	RecQ protein-like (DNA helicase Q1-like)
RECQL4	NM_004260.3	RecQ protein-like 4
RECQL5	NM_004259.6	RecQ protein-like 5
RET	NM_020975.4	Ret proto-oncogene

RFC1	NM_002913.4	Replication factor C (activator 1) 1, 145kDa
RFC2	NM_181471.2	Replication factor C (activator 1) 2, 40kDa
RFC4	NM_181573.2	Replication factor C (activator 1) 4, 37kDa
RHBDF2	NM_024599.5	Rhomboid 5 homolog 2
RNF146	NM_001242844.1	Ring finger protein 146
RNF168	NM_152617.3	Ring finger protein 168, E3 ubiquitin protein ligase
RNF8	NM_003958.3	Ring finger protein 8, E3 ubiquitin protein ligase
RPA1	NM_002945.3	Replication protein A1, 70kDa
RUNX1	NM_001754.4	Runt-related transcription factor 1
SBDS	NM_016038.2	SBDS ribosome maturation factor
SDHA	NM_004168.3	Succinate dehydrogenase complex flavoprotein subunit A
SDHAF2	NM_017841.2	Succinate dehydrogenase complex assembly factor 2
SDHB	NM_003000.2	Succinate dehydrogenase complex, subunit B, iron sulfur (Ip)
SDHC	NM_003001.3	Succinate dehydrogenase complex subunit C
SDHD	NM_003002.3	Succinate dehydrogenase complex subunit D
SETBP1	NM_002894.2	SET binding protein 1
SETX	NM_015046.5	Senataxin
SHPRH	NM_001042683.2	SNF2 histone linker PHD RING helicase, E3 ubiquitin protein ligase
SLX4	NM_032444.2	SLX4 structure-specific endonuclease subunit homolog
SMAD4	NM_005359.5	SMAD family member 4

SMARCA4	NM_001128849.1	SWI/SNF related, matrix associated, actin dependent regulator of chromatin, subfamily a, member 4
SMARCB1	NM_003073.4	SWI/SNF related, matrix associated, actin dependent regulator of chromatin, subfamily b, member 1
SMARCE1	NM_003079.4	SWI/SNF related, matrix associated, actin dependent regulator of chromatin, subfamily e, member 1
SUFU	NM_016169.3	Suppressor of fused homolog
TCL1A	NM_021966.2	T-cell leukemia/lymphoma 1A
TELO2	NM_016111.3	TEL2, telomere maintenance 2, homolog
TERF2	NM_005652.4	Telomeric repeat binding factor 2
TERT	NM_198253.2	Telomerase reverse transcriptase
TLR2	NM_003264.4	Toll-like receptor 2
TLR4	NM_138554.4	Toll-like receptor 4
TMEM127	NM_017849.3	Transmembrane protein 127
TOPBP1	NM_007027.3	Topoisomerase (DNA) II binding protein 1
TP53BP1	NM_133487.3	Tumor protein p53 binding protein 1
TSC1	NM_000368.4	Tuberous sclerosis 1
TSC2	NM_000548.4	Tuberous sclerosis 2
TSHR	NM_000369.2	Thyroid stimulating hormone receptor
UBE2A	NM_003336.3	Ubiquitin-conjugating enzyme E2A
UBE2B	NM_003337.3	Ubiquitin-conjugating enzyme E2B
UBE2I	NM_003345	Ubiquitin-conjugating enzyme E2I
UBE2V2	NM_003350.2	Ubiquitin-conjugating enzyme E2 variant 2
UBE4B	NM_001105562.2	Ubiquitination factor E4B
UIMC1	NM_001199297.2	Ubiquitin interaction motif containing 1

VHL	NM_000551.3	Von Hippel-Lindau tumor suppressor, E3 ubiquitin protein ligase
WRN	NM_000553.4	Werner syndrome, RecQ helicase-like
WT1	NM_024426.4	Wilms tumor 1
XPA	NM_000380.3	Xeroderma pigmentosum, complementation group A
XPC	NM_004628.4	Xeroderma pigmentosum, complementation group C
XRCC1	NM_006297.2	X-ray repair complementing defective repair in Chinese hamster cells 1
XRCC2	NM_005431.1	X-ray repair complementing defective repair in Chinese hamster cells 2
XRCC3	NM_005432.3	X-ray repair complementing defective repair in Chinese hamster cells 3
XRCC4	NM_022406.3	X-ray repair complementing defective repair in Chinese hamster cells 4
XRCC5	NM_021141.3	X-ray repair complementing defective repair in Chinese hamster cells 5
XRCC6	NM_001469.4	X-ray repair complementing defective repair in Chinese hamster cells 6
ZNF350	NM_021632.3	Zinc finger protein 350
ZNF365	NM_014951.2	Zinc finger protein 365

[a]Only deletions causing inactivation of *MSH2* were considered. EC, endometrial cancer; N, number.

Table SIII. List of all carriers of pathogenic variant in EC-predisposition genes, including their clinical information.

Gene groups	Gene	Variant	Age at EC diagnosis, years	Histology	Fulfilled LS criteria	Fulfilled HBOC criteria	ClinVar ID
LS	*MLH1*	c.350C>T, p.(Thr117Met)	46	Endometrioid	Yes	Yes	17094
LS	*MLH1*	c.677G>A, p.(Arg226Gln)	60	Endometrioid	Yes	No	90318
LS	*MLH1*	c.790+1G>A, p.(?)	58	Endometrioid	No	No	90356
LS	*MLH1*	c.1489dup, p.(Arg497ProfsTer6)	55	Endometrioid	Yes	No	89753
LS	*MLH1*	Deletion of exons 1-13	50	Endometrioid	Yes	No	[a]
LS	*MSH2*	c.1500dup, p.(Arg501GlnfsTer12)	46	Endometrioid	Yes	Yes	90683
LS	*MSH2*	c.1720del, p.(Gln574ArgfsTer16)	45	Unknown	Yes	No	90761
LS	*MSH2*	c.2459-2_2472del, p.(Gly820fs)	51	Unknown	Yes	Yes	2430176
LS	*MSH2*	Deletion of exons 1-8	29	Endometrioid	Yes	No	[a]
LS	*MSH2*	Deletion of exons 1-16	32	Endometrioid	Yes	No	[a]
LS	*MSH2*	Deletion of exons 3	42	Endometrioid	Yes	No	[a]
LS	*MSH2*	Deletion of exons 5-6	54	Unknown	Yes	No	[a]
LS	*MSH6*	c.643del, p.(Val215Ter)	67	Endometrioid	No	No	479909
LS	*MSH6*	c.741dup, p.(Arg248ThrfsTer8)	55	Unknown	Yes	No	410494
LS	*MSH6*	c.885dup, p.(Val296SerfsTer16)	73	Endometrioid	Yes	No	641585

LS	*MSH6*	c.964_967del, p.(Ala322ProfsTer15)	62	Endometrioid/clear cell	No	No	2430174
LS	*MSH6*	c.1444C>T, p.(Arg482Ter)	65	Endometrioid	No	No	89194
LS	*MSH6*	c.1754T>C, p.(Leu585Pro)	53	Endometrioid/serous	No	No	89220
LS	*MSH6*	c.1754T>C, p.(Leu585Pro)	43	Endometrioid	Yes	No	89220
LS	*MSH6*	c.2348_2349del, p.(Cys783Ter)	45	Endometrioid/clear cell	Yes	No	89274
LS	*MSH6*	c.2677_2678del, p.(Leu893AlafsTer6)	42	Endometrioid	Yes	No	821660
LS	*MSH6*	c.2759del, p.(Lys920ArgfsTer25)	51	Unknown	Yes	No	428433
LS	*MSH6*	c.3261del, p.(Phe1088SerfsTer2)	62	Endometrioid	Yes	No	89363
LS	*MSH6*	c.3261dup, p.(Phe1088LeufsTer5)	49	Endometrioid	Yes	Yes	89364
LS	*MSH6*	c.3995T>G, p.(Leu1332Ter)	60	Endometrioid	Yes	No	2430173
LS/HBOC	*MLH1/BRCA1*	c.1483dup, p.(His495ProfsTer29)/c.5266dup, p.(Gln1756ProfsTer74)	48	Endometrioid	Yes	Yes	2430175/17677
LS/HBOC	*MSH2/ATM*	c.1676del, p.(Leu559Ter)/c.8147T>C p.(Val2716Ala)	53	Unknown	Yes	No	90743/142700
HBOC	*ATM*	c.601C>T, p.(Gln201Ter)	78	Endometrioid	No	No	265611
HBOC	*ATM*	c.2921+1G>A, p.(?)	55	Endometrioid	No	No	141182
HBOC	*ATM*	c.3250C>T, p.(Gln1084Ter)	63	papillary serous	Yes	Yes	665551
HBOC	*ATM*	Deletion of exons 35-44	70	Unknown	No	No	[a]

HBOC	*BARD1*	c.1690C>T, p.(Gln564Ter)	28	Endometrioid	Yes	Yes	127720
HBOC	*BRCA1*	c.68_69del, p.(Glu23ValfsTer17)	N.A.	Endometrioid	Yes	No	17662
HBOC	*BRCA1*	c.1687C>T, p.(Gln563Ter)	80	EIN	No	No	37426
HBOC	*BRCA1*	c.2411_2412del, p.(Gln804LeufsTer5)	53	EIN	Yes	Yes	37466
HBOC	*BRCA1*	c.5095C>T, p.(Arg1699Trp)	69	Endometrioid	Yes	Yes	55396
HBOC	*BRCA1*	c.5510G>A, p.(Trp1837Ter)	N.A.	Unknown	Yes	No	55608
HBOC	*BRCA1*	c.5266dup, p.(Gln1756ProfsTer74)	61	Unknown	Yes	Yes	17677
HBOC	*BRCA1*	c.5266dup, p.(Gln1756ProfsTer74)	68	Leiomyosarcoma	No	Yes	17677
HBOC	*BRCA1*	c.5266dup, p.(Gln1756ProfsTer74)	N.A.	Unknown	Yes	Yes	17677
HBOC	*BRCA1*	c.5266dup, p.(Gln1756ProfsTer74)	41	Papillary serous	Yes	Yes	17677
HBOC	*BRCA1*	Deletion of exons 1-17	57	Endometrioid	No	Yes	[a]
HBOC	*BRCA2*	c.658_659del, p.(Val220IlefsTer4)	N.A.	Unknown	No	No	9342
HBOC	*BRCA2*	c.2808_2811del, p.(Ala938ProfsTer21)	41	Unknown	Yes	No	9322
HBOC	*BRCA2*	c.5213_5216del, p.(Thr1738IlefsTer2)	50	Endometrioid	No	No	37951
HBOC	*BRCA2*	c.5946del, p.(Ser1982ArgfsTer22)	55	Endometrioid	No	No	9325
HBOC	*BRCA2*	c.6275_6276del, p.(Leu2092ProfsTer7)	57	Endometrioid	No	Yes	9318
HBOC	*BRCA2*	c.6591_6592del, p.(Glu2198AsnfsTer4)	62	Unknown	No	No	9319
HBOC	*BRCA2*	c.7878G>C, p.(Trp2626Cys)	63	Leiomyosarcoma	No	No	38125
HBOC	*BRIP1*	c.2038_2039dup, p.(Leu680PhefsTer9)	71	Leiomyosarcoma	No	No	128166
HBOC	*CHEK2*	c.444+1G>A, p.(Gln149IlefsTer6)	62	Endometrioid	No	No	128075
HBOC	*CHEK2*	c.444+1G>A, p.Gln149IlefsTer6	37	Unknown	Yes	Yes	128075

HBOC	*CHEK2*	c.846+4_846+7del, p.Asp265_His282del	64	Endometrioid	No	No	216652
HBOC	*CHEK2*	c.846+4_846+7del, p.Asp265_His282del	65	Unknown	Yes	No	216652
HBOC	*CHEK2*	c.846+4_846+7del, p.Asp265_His282del	44	Endometrioid	Yes	No	216652
HBOC	*CHEK2*	c.894T>G, p.(Tyr298Ter)	61	Unknown	Yes	No	232048
HBOC	*PALB2*	c.761C>G, p.(Ser254Ter)	53	Leiomyosarcoma	Yes	No	632930
HBOC	*PTEN*	c.170T>G, p.(Leu57Trp)	57	Unknown	Yes	No	185713
HBOC	*RAD51C*	c.502A>T, p.(Arg168Ter)	62	Endometrioid	Yes	Yes	141095
HBOC	*RAD51C*	c.1026+5_1026+7del	34	Unknown	Yes	Yes	128201

[a]Copy number variations were not submitted to ClinVar because the exact nucleotide coordinates of the breakpoints required for the ClinVar submission were not determined. EC, endometrial cancer; EIN, endometrial intraepithelial neoplasia; HBOC, hereditary breast and ovarian cancer; LS, Lynch syndrome; N.A., not available.

Table SIV. Frequencies of germline PV in 207 candidate genes.

Germline PV	Indication for germline genetic testing				All EC-predisposition gene-negative patients, n (%) (N=467)	EC-predisposition gene-negative PMC, n (%) (N=1616)	All patients with EC vs. PMC	
	LS, n (%) (N=126)	HBOC, n (%) (N=13)	LS + HBOC, n (%) (N=67)	Non-indicated, n (%) (N=261)			OR (95% CI)	P-value
ATRIP[a]	0	0	1[a] (1.5)	1 (0.4)	2[a] (0.4)	3 (0.2)	2.3 (0.4-13.9)	0.3
CASP8	0	0	1 (1.5)	0	1 (0.2)	1 (0.1)	3.2 (0.2-50.5)	0.4
CWF19L2	0	0	0	0	0	2 (0.1)	N.A.	
DMBT1[b,c]	0	1[b] (7.7)	1[c] (1.5)	1 (0.4)	3[b,c] (0.6)	1 (0.1)	9.5 (1-91.5)	0.1
EMSY	0	0	0	1 (0.4)	1 (0.2)	0	N.A.	
EPHX1	1 (0.8)	0	0	0	1 (0.2)	2 (0.1)	1.6 (0.1-17.4)	0.7
ERCC3[d,e]	0	0	1[d] (1.5)	1[e] (0.4)	2[d,e] (0.4)	7 (0.4)	1 (0.2-4.7)	>0.9
ERCC4[f]	0	0	1[f] (1.5)	0	1[f] (0.2)	2 (0.1)	1.6 (0.1-17.4)	0.7
ERCC5	0	0	0	1 (0.4)	1 (0.2)	0	N.A.	
ERCC6[e]	0	0	1 (1.5)	2[e] (0.8)	3[e] (0.6)	0	N.A.	
EXO1	2 (1.6)	0	1 (1.5)	0	3 (0.6)	2 (0.1)	4.8 (0.8-28.5)	0.1
FAN1	1 (0.8)	0	0	1 (0.4)	2 (0.4)	3 (0.2)	2.3 (0.4-13.9)	0.3
FANCA	2 (1.6)	0	0	2 (0.8)	4 (0.9)	10 (0.6)	1.3 (0.4-4)	0.7
FANCD2	1 (0.8)	0	0	0	1 (0.2)	0	N.A.	
FANCE	1 (0.8)	0	0	0	1 (0.2)	1 (0.1)	3.2 (0.2-50.5)	0.4
FANCG	0	0	0	1 (0.4)	1 (0.2)	1 (0.1)	3.2 (0.2-50.5)	0.4

FANCI	1 (0.8)	0	0	0	1 (0.2)	7 (0.4)	0.5 (0.1-3.7)	0.7
FANCL[g]	0	0	1[g] (1.5)	0	1[g] (0.2)	0	N.A.	
FANCM	1 (0.8)	0	0	1 (0.4)	2 (0.4)	3 (0.2)	2.3 (0.4-13.9)	0.3
KCNJ5	0	0	0	1 (0.4)	1 (0.2)	1 (0.1)	3.2 (0.2-50.5)	0.4
LIG3	0	0	1 (1.5)	1 (0.4)	2 (0.4)	4 (0.2)	1.6 (0.3-8.6)	0.6
LRIG1	0	0	0	1 (0.4)	1 (0.2)	0	N.A.	
MCPH1	0	0	0	2 (0.8)	2 (0.4)	7 (0.4)	1 (0.2-4.7)	>0.9
MEN1	1 (0.8)	0	0	0	1 (0.2)	0	N.A.	
MLH3	0	0	0	1 (0.4)	1 (0.2)	1 (0.1)	3.2 (0.2-50.5)	0.4
MRE11A	0	0	0	1 (0.4)	1 (0.2)	0	N.A.	
MUTYH[d]	0	0	2[d] (3)	3 (1.1)	5[d] (1.1)	18 (1.1)	0.9 (0.3-2.4)	0.8
NBN[h]	2[h] (1.6)	0	0	0	2[h] (0.4)	7 (0.4)	1.0 (0.2-4.7)	>0.9
PIK3CG	0	0	0	0	0	0	N.A.	
POLD1[g]	0	0	1[g] (1.5)	0	1[g] (0.2)	0	N.A.	
POLE	0	0	1 (1.5)	1 (0.4)	2 (0.4)	1 (0.1)	6.3 (0.6-69.8)	0.1
PREX2	0	0	0	0	0	1 (0.1)	N.A.	
PRF1[a]	0	0	1[a] (1.5)	0	1[a] (0.2)	1 (0.1)	3.2 (0.2-50.5)	0.4
RAD1	0	0	1 (1.5)	0	1 (0.2)	0	N.A.	
RAD17[b,c]	0	1[b] (7.7)	1[c] (1.5)	0	2[b,c] (0.4)	2 (0.1)	3.2 (0.4-22.5)	0.2
RAD50	0	0	0	0	0	8 (0.5)	N.A.	
RAD51B	0	0	0	1 (0.4)	1 (0.2)	0	N.A.	

RAD54L[f]	0	0	1[f] (1.5)	0	1[f] (0.2)	1 (0.1)	3.2 (0.2-50.5)	0.4
RBBP8	0	0	0	1 (0.4)	1 (0.2)	0	N.A.	
RCF4[h]	1[h] (0.8)	0	0	0	1[h] (0.2)	2 (0.1)	1.6 (0.1-17.4)	0.7
RECQL	0	1 (7.7)	0	0	1 (0.2)	1 (0.1)	3.2 (0.2-50.5)	0.4
RECQL4	0	0	1 (1.5)	0	1 (0.2)	8 (0.5)	1.1 (0.2-5.2)	>0.9
RECQL5	0	0	0	0	0	6 (0.4)	N.A.	
RNF168	1 (0.8)	0	0	0	1 (0.2)	1 (0.1)	3.2 (0.2-50.5)	0.4
SBDS	0	0	0	1 (0.4)	1 (0.2)	21 (1.3)	0.3 (0.1-1.3)	0.1
SETX	0	0	0	1 (0.4)	1 (0.2)	1 (0.1)	3.2 (0.2-50.5)	0.4
SLX4	0	0	0	1 (0.4)	1 (0.2)	1 (0.1)	3.2 (0.2-50.5)	0.4
XRCC4	0	0	1 (1.5)	1 (0.4)	2 (0.4)	1 (0.1)	6.3 (0.6-69.8)	0.1
All PV	15	3	19	29	66	139		
All carriers	14[h] (11.1)	2[b] (15.4)	14[a,c,d,f,g] (20.9)	28[e] (10.7)	58[a-h] (12.4)	139 (8.6)		

Pathogenic variants in two candidate cancer susceptibility genes: [a]ATRIP/PRF1, [b]DMBT1/RAD17, [c]DMBT1/RAD17, [d]ERCC3/MUTYH, [e]ERCC3/ERCC6, [f]ERCC4/RAD54L, [g]FANCL/POLD1, [h]NBN/RCF4. Frequencies of germline PV found in a subgroup of patients fulfilling criteria for germline genetic testing for LS, HBOC, LS and HBOC, individuals not fulfilling any criteria (non-indicated), an aggregated group of all patients with EC, and a group of PMC. CI, confidence interval; EC, endometrial cancer; HBOC, hereditary breast and ovarian cancer; LS, Lynch syndrome; N, number; N.A., not available; OR, odds ratio; PMC, population-matched controls; PV, pathogenic variant.
